# Supplementary figures and images for: Tracing back variations in archaeal ESCRT-based cell division to protein domain architectures
Source: PLoS One. 2022 Mar 31;17(3):e0266395. doi: 10.1371/journal.pone.0266395 (PMC8970359; doi:10.1371/journal.pone.0266395)

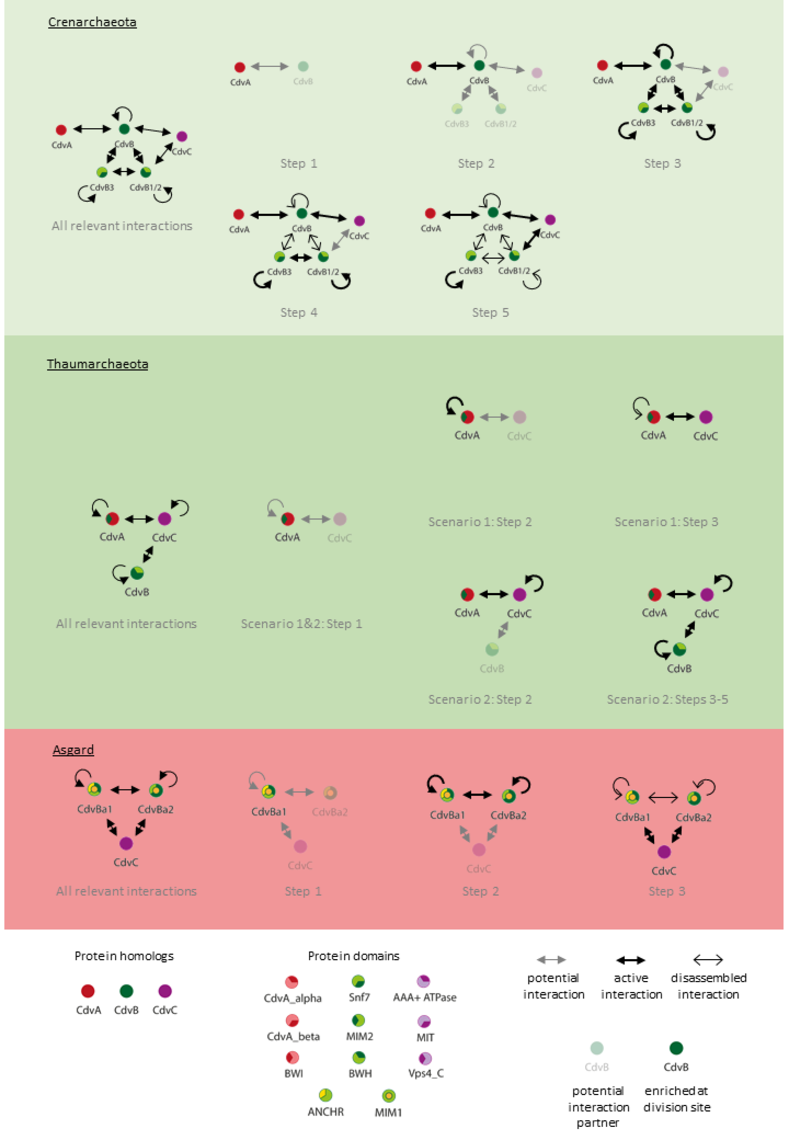

Supplement: S1 Fig — In Crenarchaeota, the potential polymerisation capabilities of CdvA and CdvC are not displayed as they do not help explain the suggested mechanism. In Asgard archaea, the CdvC polymerisation capability is likewise not displayed. Background colours indicate phylogenetic groups. Red: Asgard archaea, dark green: Thaumarchaeota, pale green: Crenarchaeota. (TIF) [file pone.0266395.s001.tif]

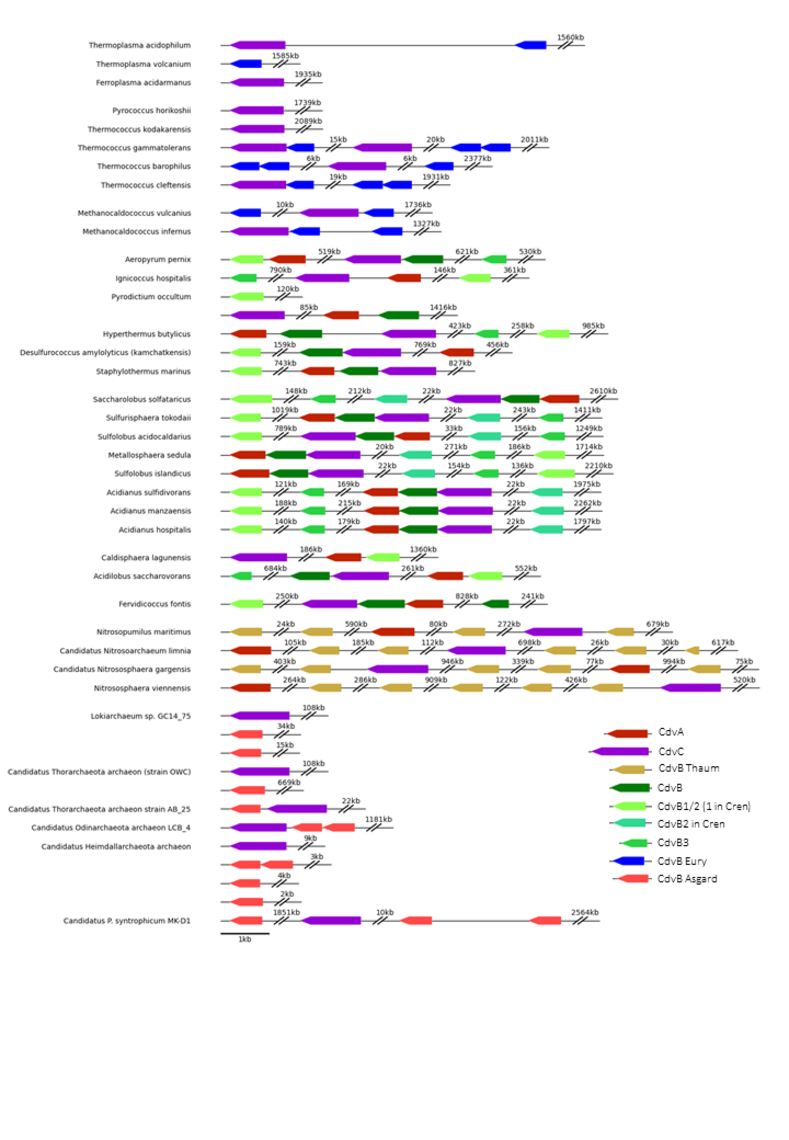

Supplement: S2 Fig — Organisms are grouped by phylogeny. Genes encoding CdvA, CdvB and CdvC are in direct neighbourhood in Crenarchaeota. In Thaumarchaeota, in three out of four organisms one CdvB homolog gene is in close neighbourhood to the CdvC gene. CdvA homologs are not. In Euryarchaeota, if there are genes encoding both CdvB and CdvC homologs, they are either in direct or in relatively close neighbourhood. In Asgard archaea, except in Cand. P. syntrophicum only fragmentary genomes are available. In three out of six Asgard archaea genomes CdvC and CdvB homolog genes are in close neighbourhood. This pattern across all archaeal phyla indicates that indeed CdvC and one CdvB homolog existed in the last common ancestor of TACK, Asgard and Euryarchaeota. (TIF) [file pone.0266395.s002.tif]

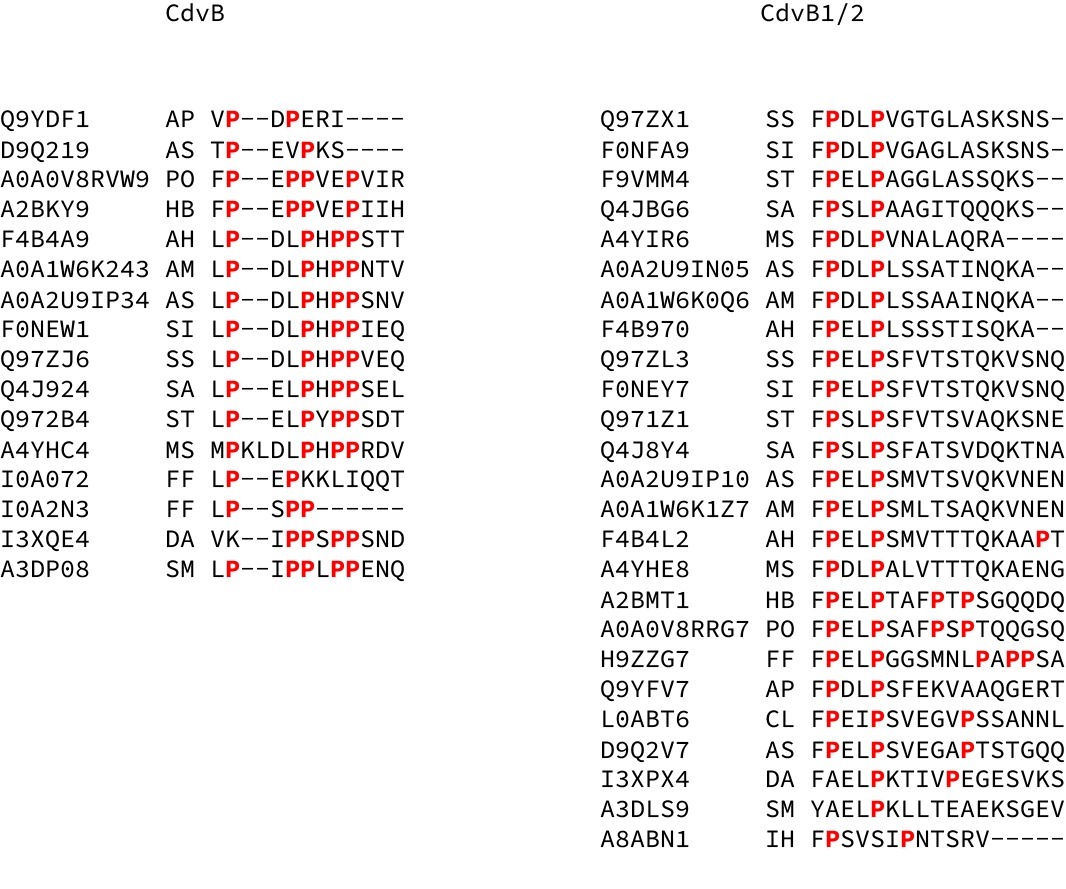

Supplement: S3 Fig — In CdvB1/2 proteins the MIM2 domain contains consistently fewer prolines, indicating a weaker affinity to the MIT domain than the MIM2 of CdvB proteins. Abbreviations: AP: Aeropyrum pernix, AS: Acidilobus saccharovorans, PO: Pyrodictium occultum, HB: Hyperthermus butylicus, AH: Acidianus hospitalis, AM: Acidianus manzaensis, AS: Acidianus sulfidivorans, SI: Sulfolobus islandicus, SS: Saccharolobus solfataricus, SA: Sulfolobus acidocaldarius, ST: Sulfurisphaera tokodaii, MS: Metallosphaera sedula, FF: Fervidicoccus fontis, DA: Desulfurococcus amylolyticus, SM: Staphylothermus marinus, CL: Caldisphaeralagunensis, IH: Ignicoccus hospitalis. (TIF) [file pone.0266395.s003.tif]

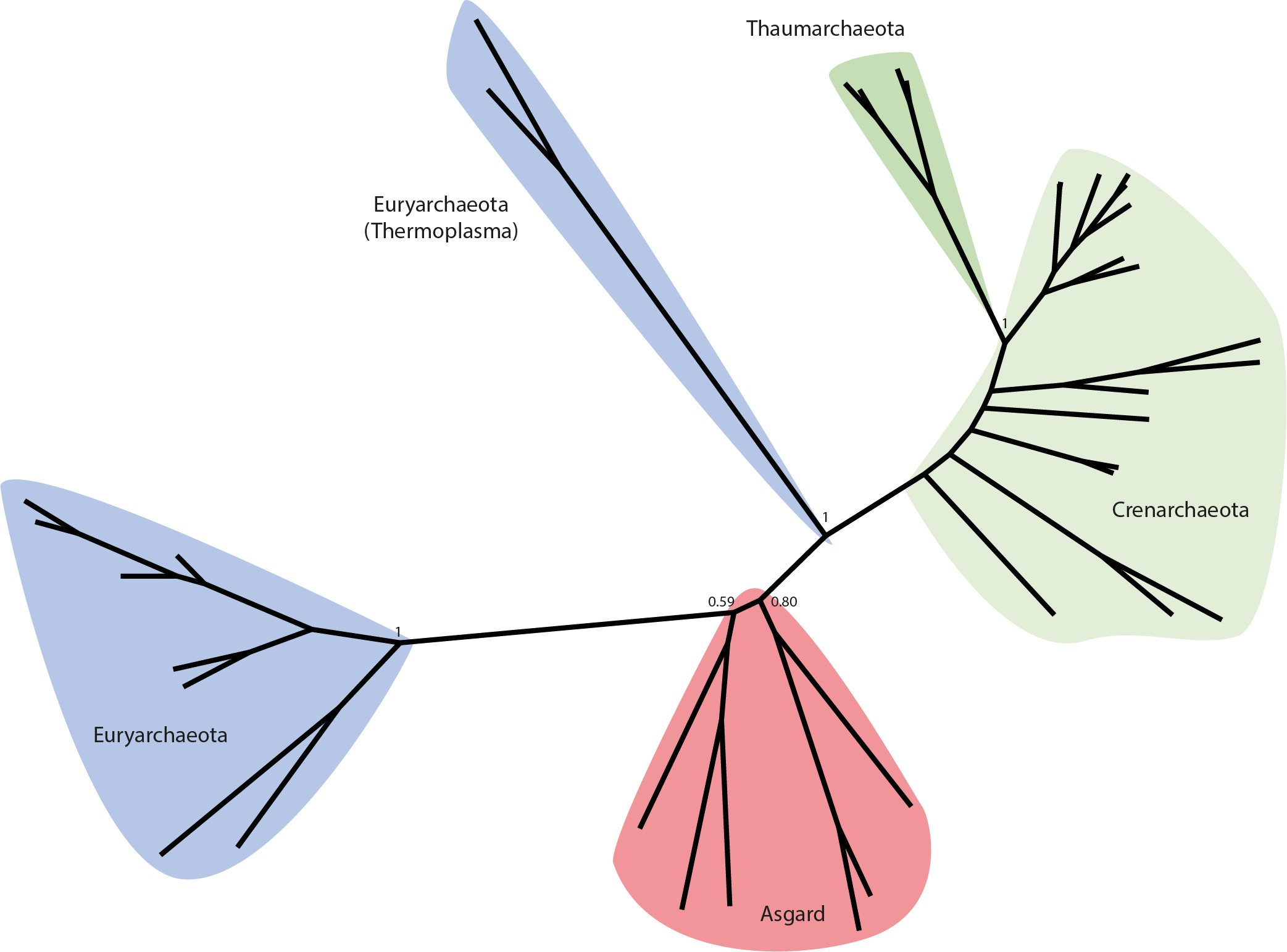

Supplement: S4 Fig — The overall structure shows the same phylogenetic relations as the CdvB tree (Fig 2): Asgard archaea are located between Euryarchaeota and Crenarchaeota, while Thaumarchaeota are most distant from Asgard archaea. However, the Euryarchaeota group of Thermoplasma is not located near all other Euryarchaeota but between Asgard archaea and TACK archaea. This might be an artifact because the proteins in Thermoplasma mutated massively and maybe gained an entirely new function (the branch is the longest in the whole phylogeny), or it might be caused by horizontal gene transfer. Background colours indicate phylogenetic groups. Blue: Euryarchaeota, red: Asgard archaea, dark green: Thaumarchaeota, pale green: Crenarchaeota. Numbers indicate posterior probabilities. (TIF) [file pone.0266395.s004.tif]
