# Supplementary material for: Tracing back variations in archaeal ESCRT-based cell division to protein domain architectures
Source: PLoS One. 2022 Mar 31;17(3):e0266395. doi: 10.1371/journal.pone.0266395 (PMC8970359; doi:10.1371/journal.pone.0266395)
Supplement: Attachment — Submitted filename: Response to Reviewers.pdf [file pone.0266395.s007.pdf]

Response to Reviewers

We want to thank the reviewers for their fair, valuable, and remarkably detailed feedback. They raised valid points that helped us to identify weaknesses of our argumentation and communication. We believe that the revised manuscript profits significantly from the reviewers remarks and now is a much more understandable and enjoyable read.

Reviewer #1

The greatest problem emerges in sentences like:

142

230-234

318-321

where, despite the fact that it is entirely bioinformatic study, additional analyses are required to allow consolidation of their considerations

We are unsure what the reviewer means with that comment. We believe that we have made sufficiently clear that the intention of this non-experimental paper is to inform experimentalists about sensible and promising experimental approaches based on our thorough bioinformatic analysis. Obviously, to consolidate our data-based hypotheses, experimental evidence will be required. We changed the sentences mentioned by the reviewer slightly where necessary by emphasizing that these are hypotheses that we hope will inspire experimentalists.

In 131: They selected 37 organisms for their analysis. But in Figure 1 and Suppl. Table 1. The set of organisms that they present corresponds to a set of 51 organisms.

In Figure 1 and Suppl. Table 1, 51 organisms are displayed, out of which 37 possess at least one CdvABC homolog. Thus, CdvABC homologs from these 37 organisms were further analysed. The other 14 organisms are merely displayed in Figure 1 to visualise that in Euryarchaeota many organisms do not possess CdvABC homologs, which is relevant because it is thought that Euryarchaeota divide by a mechanism not utilising Cdv. If a reader looks at Figure 1, s:he should instantaneously see that in Euryarchaeota Cdv proteins are not the norm, whilst in TACK archaea they are (with the exception of Thermoproteales). This is why we included organisms not possessing CdvABC homologs in the Figure.

To make this clear that we selected 37 organisms containing CdvABC homologs for further analysis, we changed the sentence at 131 to “From the resulting list of organisms containing at least one CdvABC homolog we selected 37 organisms by quality and analysed […]” and wrote in the Figure caption that out of the 51 displayed organisms 37 possess CdvABC homologs.

In 133: CDD needs to be defined the first time it is used.

We did define CDD.

In 135: Figure 1B does not contain the eleven domains that they describe in the text.

AND

In general, figure captions are very extensive and tend to repeat information from the main text. As a result, they should be reorganized in general.

In this context, Figure 1 includes too much information forcing the transfer of a component to Figure 3. It is preferable to create one more figure that makes the information more clear; this will help to better understand the phylogenetic information. Finally, the legend of the figure must be written in a sequential manner and must avoid discussing portion B) at the conclusion.

AND

Figure 3 shows the domain table with information that is duplicated from Figure 1B.

Both reviewers criticised the Figure captions and the density of information in Figure 1. We thank them for their suggestion, as we agree that the paper would be easier to understand and more enjoyable to read with simpler and less crowded Figures. Therefore, we reorganised Figures 1 and 3 such that in the revised manuscript, there are now six Figures instead of four: The new Figure 1 is a completely reworked display of the distribution of CdvABC homologs in archaea, which was Figure 1A in the old manuscript. It does not contain any other information, making it much clearer than in the previous version (as suggested by the reviewer). Parts B to E of the old Figure 1 were moved to a new Figure (4), together with Figure 3B of the old manuscript (again, as suggested by the reviewer). Thus, the new Figure 4 contains all the hypothesised mechanisms in one Figure. We also added a new Figure (2) displaying the potential protein-protein interactions in Cren-, Thaum- and Asgard archaea. We believe that this visualisation helps the reader to better understand the story we want to tell and was missing in the previous version of the manuscript.

The old Figure 4 remained the same, but is now Figure 6. The old Figure 2 is now Figure 3.

Furthermore, with the reorganisation of the Figures we also changed the Figure captions to more compact texts as suggested by both reviewers.

They indicate that CDv genes are identified in just a few species in the case of Euryarchaeota. It would be interesting to study if these remaining genes have been subjected to relaxed selection pressures. To back up the decision expressed in lines 200-202.

We thank the reviewer for this suggestion. In section “Cdv protein domains in Euryarchaeota do not constitute a common functional system” we included a short description and references to recent experimental studies indicating that Euryarchaeota use a FtsZ-based cell division machinery instead of a Cdv-based machinery, and relate this to our observation that only in few Euryarchaeota we found Cdv genes. We also refer to Suppl. Table 2, where we included FtsZ homologs (this data was already in the previous version of Suppl. Table 2, but not referenced to in the main text).

In 238, change T to t.

We did as suggested by the Reviewer.

If the interactions described are previously known, the section 230-242 should be appropriately cited, and if they are the results of the study, further proof of these interactions should be provided.

We agree that this section could have caused confusions. We reworked the entire section "Protein domain compositions explain experimental findings indicating differences between Cren- and Thaumarchaeota" and created a new Figure (Figure 2) to properly explain our approach and appropriately cite the experiments interactions were inferred from.

Regarding figure 2, it is unclear if the phylogeny was constructed using the 37 organisms or the 51 in figure 1, and how the organisms in the Eukaryotic clades were chosen. Check the Eukaryotic typeface in figure 2.

The phylogeny was constructed using a multiple alignment of all CdvB/ESCRT-III homologs. It is a protein tree. Thus, every leave in the tree represents one protein. Out of the 51 organisms in Figure 1, 37 organisms possess CdvB homologs. Thus, proteins from these organisms were selected to construct the phylogeny. The phylogeny displays how the *proteins* are related, which does not necessarily reflect the phylogeny of the organisms. Most of the 37 organisms possess multiple paralogs, which were all included in the phylogeny. The total number of archaeal proteins used to construct the phylogeny after all was 100 (all CdvB homologs included in this study). We changed the legend of Figure 2 (now Figure 3, see above) to explain this better.

Although the article does not focus on other types of machinery, it would be preferable for the authors to address more fully what happens with organisms that do not conserve CDv genes.

To provide the reader some information about cell division in archaea without Cdv systems, we included a short description that Euryarchaeota use a FtsZ-based cell division machinery in the section "Cdv protein domains in Euryarchaeota do not constitute a common functional system", together with information about FtsZ homologs in Suppl. Table 2, and mention that Thermoproteales were suggested to utilise an actin-based cell division system in the section "Cdv proteins of Cren- and Thaumarchaeota are composed of different domains". Furthermore, to make clear to the reader from the beginning on that this article is about Cdv/ESCRT-based cell division only, not about cell division in archaea in general, we changed the title of the article to "Tracing back variations in archaeal ESCRT-based cell division to protein domain architectures".

Finally, all data, including scripts, should be included as supplemental material or deposited in a freely accessible repository.

We uploaded our data to a public github repository (https://github.com/BelaFrohn/ArchaealESCRTDomains).

Reviewer #2

However, I would like to ask for the authors to create and share with us a robust and reproducible (bioinformatic) algorithm or tool (script) to identify Archaeal CdvABC proteins. While reading their methods it sometimes feels like they are cherry-picking some protein sequences and discarding others.

That is only the feeling I get when reading this version of the manuscript and I would greatly appreciate something like a validated HMM that could be offered to the community for these Archaeal proteins.

While we agree that a bioinformatic tool to identify archaeal CdvABC proteins might be a valuable tool for Archaea researchers, this was not the goal of our study and we do not believe that we could better communicate the ideas of our manuscript with the help of such a tool. The goal of our study was not to describe or analyse a variety of Cdv-based archaeal cell division on a single organism level, but to find and describe common principles on a larger scale. For our analysis we used protein sequences that were previously labeled as CdvABC homologs in the eggNOG database, which we believe is trustworthy, and sequences that were used in a similar phylogenetic analysis by Makarova et al. (Makarova KS, Yutin N, Bell SD, Koonin E V. Evolution of diverse cell division and vesicle formation systems in Archaea. Nat Rev Microbiol. 2010). By comparing our Figure 1 to Figure 2 of Makarova et al., one can see that while we included more TACK organisms in our study, the homolog composition in our study and theirs is the same, reassuring the correctness of our protein selection. Furthermore, they included several more organisms from Euryarchaeota, and the result that only very few of them possess CdvABC homologs is the same as in our study, again reassuring our analysis. Thus, we are convinced that we did not fall into the trap of cherry-picking and that including more organisms – with less trustworthy sequence data - in our study would only distract the reader from the rather general statements about archaeal cell division we want to communicate.

Are the assignments of the protein domains described in the article similar to what tools like InterPro scan reveal as output using HMMs? and, if no Pfam model were available. would it be possible to obtain the same results when creating HMMs using your set of known domains instead of regular expressions?

I would ultimately like to know if your approach is as sensitive and/or robust as the extensive use of HMMs to determine a proteomic domain or motif.

If we understand the reviewer correctly, s:he refers to the identification of the MIM1, MIM2 and ANCHR motifs (other domains were identified by InterProScan as described by the reviewer). The problem we had to deal with when identifying these motifs was that (a) the motifs are very short, (b) they are defined by the describing authors via regular expressions and (c) there are only few sequences where these motifs are clearly described. Thus, we decided to use regular expression search, as this matched the approach used by the describing authors best. We believe that it is only consequential to search motifs that are defined by regular expressions via regular expressions. To directly answer the question of the reviewer, as we did not do it, we do not know how a HMM would have performed on these short motifs. However, we believe that constructing and training HMMs for these motifs would have been problematic, given the shortness of the motifs and the little amount of trustworthy data available.

Resolution of the Figures I reviewed was very low and I hope that this is due to the Plos review platform and not the authors' design. Figures 1 and 3 are a vital component of this article and should have exceptional resolution to offer the most needed aid the reader requires to understand the different variations of the CdvABC secondary structures and their proposed mechanisms.

We uploaded the Figure in high resolution.

Please note that major parts of the "Figure legends" should be moved to the "Results" or "Discussion" sections of the main text as descriptions of the outcome of your analysis, and not as the caption describing the figure to the reader.

AND

In Figure 1A legend please move to the Results section descriptions of the outcome of your analysis, such as those from line no. 163 "Differing domain patterns matching the phylogenetic groups are clearly visible…" to line no. 177. There are more phrases further below that can also be moved to results instead of the figure caption.

AND

Text from line 276 to 286 should be incorporated to the "Results" or "Discussion" sections of the main text as descriptions of the outcome of your analysis

AND

Text from line 399 to 406 should be incorporated to the "Results" or "Discussion" sections of the main text as descriptions of the outcome of your analysis

AND

Text from line 461 to 467 should be incorporated to the "Results" or "Discussion" sections of the main text as descriptions of the outcome of your analysis

We thank the reviewer for the suggestion and entirely reorganised our Figures. We designed 3 new Figures and overhauled the captions based on the suggestions of both the reviewers. See above at Reviewer #1 for more details.

In line no. 14 "... This spread across super-phyla suggests that…" Archaea and Eukaryotes have the taxonomic rank of "Domains" of life, not "Super-phyla", specify that you assessed "super-phyla within these Domains".

We changed the term, as suggested by the reviewer, resulting in minor changes in the Summary.

In line no. 68 "... After the discovery of CdvA, -B and -C in S. acidocaldarius, …" Better refer to these three proteins as "CdvABC" here and below.

We did as suggested by the reviewer. We adopted a nomenclature where if referring explicitly to all three proteins CdvA, CdvB and CdvC together, we write CdvABC, whilst if referring to a machinery using Cdv proteins, we write Cdv.

In line no. 82 "... concept as one highly refined version of the Asgard Cdv system. This relationship emphasizes that it is necessary to investigate archaeal Cdv machineries in addition to eukaryotic ESCRT machineries to draw conclusions about the ancestral machinery."

This phrase would read better if written something like:

We were not sure what changes the reviewer wishes, but we rephrased the ending of the paragraph to (hopefully) be easier to read and understand.

In line no. 101 "... On the one hand, experimental studies with archaeal Cdv machineries are technically highly challenging, due to the extreme conditions archaeal organisms thrive under. On the other

hand,..." These phrases would read better if you started them with "First" and Second" instead of "on one hand and on the other".

We rephrased the sentence as suggested.

In line no. 150 "... Thus, this simple analysis already yielded two interesting results: First, four domains are shared between all organisms…" Please constrain the extent of your findings to "all genomes included in this analysis" instead of "all organisms".

We restrict ourselves to "organisms included in this study" in the revised manuscript.

In line no. 161 I recommend a more universal color name instead of "Khaki", such as "yellow" or "pale yellow".

We use only simple colour names in the revised manuscript.

248 "Thus, these previously unexplained experimental differences fit nicely to our analysis of potential PPIs." I suggest changing "fit nicely" with "are further supported by" or something similar.

We did as suggested by the reviewer.

Please rephrase line no. 249 "potential PPIs. Furthermore, it was observed that in Crenarchaeota it is indeed the CdvB homolog with the BWH domain that enriches first, followed by the other CdvB homologs (5)."

We rephrased this and the following sentence to better explain how our suggested PPI networks are consistent with the literature.

In line no. 261 "Reassuringly, the differences in domain architecture fitted perfectly to the phylogenetic clusters (Figure 2)." change to something like "... were congruent with their phylogeny, as seen by their placement in different branches within the CdvB tree"

We toned the sentence down exactly as suggested. To remain consistent, we avoided the term "phylogenetic cluster" and used the term "branch" whenever possible subsequently.

Please tone down the following phrase (that should be in Results section and not as part of a Figure legend) to clarify that this is an hypothetical explanation derived from your data (e.g. "Our data supports that…"): in line no. 277 "This means that all interactions between different CdvB homologs developed independently and are therefore not comparable."

The sentence was dropped during the reworking of the figure legend.

Please change line no. 380 “Our analysis of multiple Asgard archaea genomes and especially the trustworthy Cand. P. synthrophicum genome…” with something like “Our analysis of multiple Asgard archaea genomes and especially that of its only cultivated member Cand. P. synthrophicum…”

and

Please change in line no. 481 “The mechanism we suggested for Crenarchaeota fits nicely to a recent study of Tarrason Risa et al. (6)” with “... further supports the recent study of…”

We did exactly as suggested by the reviewer.

In line no. 594 “... to run PSI-Blast (27) against the NCBI protein (28) database.” Please specify which NCBI database version you used (e.g. v2021-12-20)

We specified the version.

In line no. 610 “Decomposing proteins into domains” Please consider changing this to “Fragmented analysis of protein domains” or something like that (here and along all the methods section below)

We used the term “Fragmenting” instead of “Decomposing” there and subsequently and clarified that we talk about protein sequence data, not experiments on proteins.
